# Racial and Ethnic Disparities in Regulatory Air Quality Monitor Locations in the US

**DOI:** 10.1001/jamanetworkopen.2024.49005

**Published:** 2024-12-04

**Authors:** Brenna C. Kelly, Thomas J. Cova, Michelle P. Debbink, Tracy Onega, Simon C. Brewer

**Affiliations:** 1 Department of Population Health Sciences, University of Utah, Salt Lake City; 2 School of Environment, Society and Sustainability, University of Utah, Salt Lake City; 3 Departments of Obstetrics and Gynecology, University of Utah, Salt Lake City

## Abstract

**Importance:**

Understanding exposure to air pollution is important to public health, and disparities in the spatial distribution of regulatory air quality monitors could lead to exposure misclassification bias.

**Objective:**

To determine whether racial and ethnic disparities exist in Environmental Protection Agency (EPA) regulatory air quality monitor locations in the US.

**Design, Setting, and Participants:**

This national cross-sectional study included air quality monitors in the EPA Air Quality System regulatory monitoring repository, as well as 2022 American Community Survey Census block group estimates for racial and ethnic composition and population size. Bayesian mixed-effects models of the count of criteria pollutant monitors measuring an area were used, adjusting for population size and accounting for spatial autocorrelation. Data were analyzed from March to June 2024.

**Exposure:**

Census block group–level racial and ethnic composition.

**Main Outcome and Measures:**

Number of regulatory monitors measuring a census block group by criteria pollutant (particulate matter [PM], ozone [O_3_], nitrogen dioxide [NO_2_], sulfur dioxide [SO_2_], lead [Pb], and carbon monoxide [CO]).

**Results:**

This analysis included 329 725 481 individuals living in 237 631 block groups in the US (1 936 842 [0.6%] American Indian and Alaska Native, 18 554 697 [5.6%] Asian, 40 196 302 [12.2%] Black, 60 806 969 [18.4%] Hispanic, 555 712 [0.2%] Native Hawaiian and Other Pacific Islander, 196 010 370 [59.4%] White, 1 208 267 [0.3%] some other race, and 10 456 322 [0.4%] 2 or more races). Adjusting for population size, monitoring disparities were identified for each criteria pollutant. Relative to the White non-Latino population, all groups were associated with fewer NO_2_, O_3_, Pb, and PM monitors. Disparities were consistently largest for Native Hawaiian and Other Pacific Islander populations, followed by American Indian and Alaska Native populations and those of 2 or more races. An increase in percentage of Native Hawaiian and Other Pacific Islander race was associated with fewer monitors for SO_2_ (adjusted odds ratio [aOR], 0.91; 95% BCI, 0.90-0.91), CO (aOR, 0.95; 95% BCI, 0.94-0.95), O_3_ (aOR, 0.95; 95% BCI, 0.94-0.95), NO_2_ (aOR, 0.97; 95% BCI, 0.91-0.94), and PM (aOR, 0.96; 95% BCI, 0.95-0.96). An increase in the percentage of those of Asian race was associated with slightly more SO_2_ (aOR, 1.04; 95% BCI, 1.03-1.04) monitors.

**Conclusions and Relevance:**

This cross-sectional study of racial and ethnic disparities in the location of EPA regulatory monitors determined that data may not be equitably representative of air quality, particularly for areas with predominantly Native Hawaiian and Other Pacific Islander or American Indian or Alaska Native populations. Integration of multiple data sources may aid in filling monitoring gaps across race and ethnicity. Where possible, researchers should quantify uncertainty in exposure estimates.

## Key Points

**Question:**

Do Environmental Protection Agency (EPA) regulatory monitors equitably represent the US population across race and ethnicity?

**Findings:**

This cross-sectional study with 329 725 481 individuals found that monitoring disparities exist for all criteria pollutants, particularly sulfur dioxide and lead, followed by ozone and carbon monoxide. Disparities were consistent across most racial and ethnic groups but were generally largest for those of Native Hawaiian and Pacific Islander race and American Indian or Alaska Native race.

**Meaning:**

These findings suggest regulatory monitor data may not adequately capture air quality exposures for some marginalized race and ethnicity groups, and the consequences of incomplete or uncertain air quality estimates for these communities should be further investigated.

## Teaser

This cross-sectional study evaluates racial and ethnic disparities in Environmental Protection Agency regulatory air quality monitor locations in the US.

## Introduction

Air pollution creates a substantial public health burden,^1^ and understanding exposure is essential to researching, preventing, and responding to associated diseases. Data from the Environmental Protection Agency’s (EPA) regulatory monitors is a critical resource for estimating exposure to common, harmful pollutants. However, little is known about whether racial and ethnic disparities exist in the spatial distribution of these monitors. Air quality is geographically heterogenous, and monitoring disparities could result in misclassification of exposures, potentially compounding known environmental injustices.

Despite the robust body of literature describing inequities in air pollution exposure, inequity in air quality monitoring has not been a major research focus.^2,3^ While federal regulatory monitors are considered the reference standard for air quality monitoring,^4^ the EPA has acknowledged the spatial sparseness of regulatory monitors in the US.^5^ The location of a monitor is determined by both federal requirements and state, local, or tribal authoritities.^6^ Sampling bias could potentially lead to incorrect conclusions about the safety of air quality. Monitors measure a single point in space, meant to be a representative sample of a larger body of air. To estimate nearby pollutant concentrations, these point samples are usually smoothed across space using some form of interpolation. Importantly, this method cannot capture more variability than was observed; we may interpolate but not extrapolate. Furthermore, interpolating over increasing distances also increases the uncertainty of estimates in the intervening space between monitors. This uncertainty is particularly problematic when studying the effect of exposures on associated health end points such as asthma,^7^ cardiovascular disease,^8^ and all-cause mortality.^9^ If true exposures are lower or higher than estimates from monitor interpolation, we may not understand the true effect of exposure dose on disease risk.

Previous work^10^ has investigated regulatory monitor locations at the county level in the contiguous US, but only for fine particulate matter (PM_2.5_) and ozone (O_3_), and using only Black or African American race and Hispanic or Latino ethnicity. The authors found that counties with insufficient monitoring had a lower percentage of both of these groups. A more recent study of socioeconomic disparities in low-cost sensors in California found that fewer fine particulate matter monitors were located in census tracts with greater Hispanic or Latino and Black or African American populations, accounting for population density.^11^

Racial disparities in air pollution exposure are well established across the criteria pollutants,^12,13^ namely particulate matter (PM), O_3_, nitrogen dioxide (NO_2_), sulfur dioxide (SO_2_), lead (Pb), and carbon monoxide (CO). However, if monitoring disparities also exist, these observed disparities could be partially based on smoothed estimates, possibly overestimating or underestimating actual exposure. The objective of this study was to investigate racial and ethnic disparities in the location of EPA regulatory air quality monitors in the US.

## Methods

For each criteria pollutant, we used a mixed-effects negative-binomial model to investigate associations between the number of EPA monitors and block group-level racial and ethnic composition, accounting for population size and spatial autocorrelation. An institutional-determined waiver of the need for review and informed consent was approved for this study by the University of Utah institutional review board because data were deidentified. The Strengthening the Reporting of Observational Studies in Epidemiology (STROBE) reporting guideline for cross-sectional studies was used in preparing this manuscript.

### Study Design and Population

We analyzed data from the EPA Air Quality System (AQS),^14^ a repository of air quality data collected from regulatory monitors, as well as 2022 American Community Survey (ACS) 5-year estimates of racial and ethnic composition at the census block group level. Monitors that were active between March 2019 and March 2024 were included in the analysis. Census block groups for all US states and the District of Columbia were included. We used census-calculated population-weighted centroids to capture the distribution of a population within the block group.^15^

### Race and Ethnicity

The percentage of each census-defined race and ethnicity in the 2022 ACS (American Indian or Alaska Native, Asian, Black or African American, Hispanic or Latino, Native Hawaiian or Other Pacific Islander, White, 2 or more races, some other race) were collected at the census block group level using the R tidycensus package.^16^ All racial groups were collected as non-Hispanic or Latino, such that race and ethnicity categories were mutually exclusive. Variables were log-transformed due to strong right-skewed distributions.^17^ Coefficients were interpreted as a log-unit increase in the percentage of a given race or ethnicity relative to a mostly White non-Hispanic or Latino population (eAppendix 1 in Supplement 1). Maps of racial and ethnic composition are provided in eFigures 1 and 2 in Supplement 1.

### Outcome: Number of Criteria Pollutant Monitors

**Figure 1.**
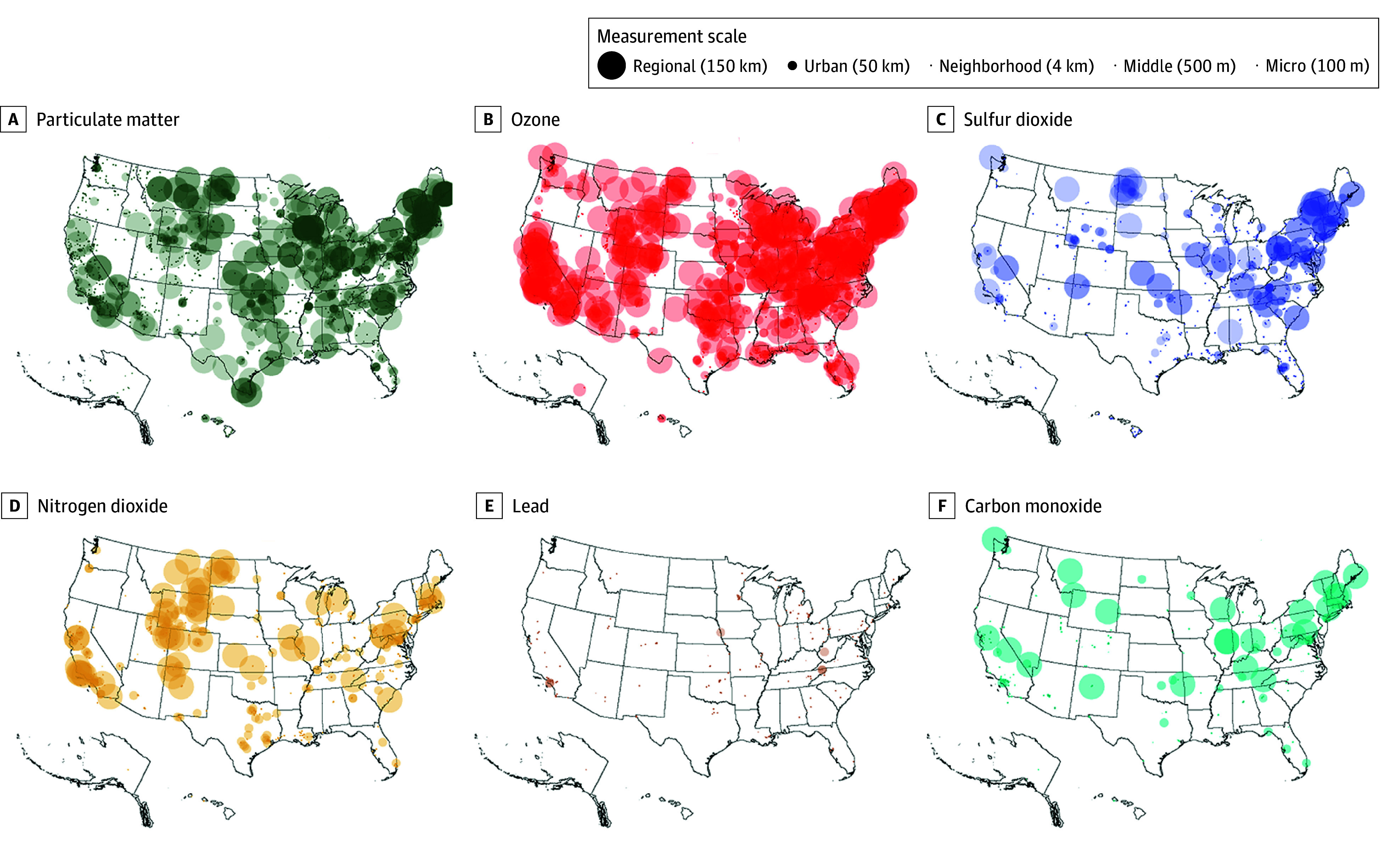
Environmental Protection Agency Regulatory Monitor Coverage by Criteria Pollutant Spatial coverage of the US is shown for each monitor by criteria pollutant.

To model the number of criteria pollutants at the census block group level, we needed to determine whether each monitor monitored a census block group. Monitors sample 1 point that is meant to be representative of a larger area. The measurement scale of a monitor, also called the spatial extent, indicates how large this area is. Microscale monitors sample up to 100 m, middle scale from 100 m to 0.5 km, neighborhood scale from 0.5 to 4 km, urban scale from 4 km to 50 km, and regional scale from tens to hundreds of kilometers.^18^ For monitors that were missing a measurement scale, the modal scale for that criteria pollutant was imputed, capturing the typical scale for each pollutant (eTable 1 in Supplement 1). Buffers representing every monitor’s scale were created (Figure 1). If a monitor’s buffer intersected with a ensus block group’s population-weighted centroid, that population was considered to be monitored by that monitor (eFigure 3 in Supplement 1). This process was performed for each criteria pollutant (PM, O_3_, NO_2_, SO_2_, Pb, and CO). The number of monitors for each criteria pollutant at the census block group level was then modeled. Only monitors that collected data within the past 5 years (2019-2024) were included. Alternative strategies for defining the outcome are described in eAppendix 2 in Supplement 1.

### Population Size

To identify potential confounding factors, we used a directed acyclic graph to delineate well-established associations among the variables under consideration in our study (eFigure 4 in Supplement 1).^19^ Population size emerged as a potential confounder in the minimal adjustment set, defined as the minimal set of variables which, when conditioned on, provided an unbiased estimate of a proposed assocation.^19^ Previous work analyzing monitor locations has also accounted for population when studying racial and ethnic composition as proportions.^11^ We computed the expected number of monitors for each pollutant (*E_i_*) measuring a block group given the population distribution. *E_i_* was included in our models as specified in the Statistical Analysis section.

### Statistical Analysis

We use bayesian mixed-effects regression models to relate the number of criteria pollutant monitors (*y_i_*) to racial and ethnic composition within census block groups (*i*). The model was specified assuming that counts are negative-binomial distributed^20^:

*y_i_ ~ Negative − binomial*(*mean* = *E_i_*θ*_i_, overdispersion* = *ω_k_*), *I* = 1, …, *n*

where *E_i_* is the expected count and θ*_i_* is the relative risk of having a monitor in area *i*. The expected rate is calculated as:

*E_i_* = *r*^(^*^m^*^)^*n_i_*

where *r*^(^*^m^*^)^ is the total number of monitors for each criteria pollutant divided by the total US population, and *n_i_* is the population of each census block group *i*. The logarithm is expressed as:

log(θ*_i_*) = α*_i_* + *βX_i_* + *u_i_* + *v_i_*

where α*_i_* is the dispersion parameter with unknown mean μ_α_ and standard deviation σ_α_. The spatial random effect is modeled using spatially structured error *u_i_*, and random error *v_i_*. The Besag-York-Mollié model^21^ is used to specify *u_i_* with a conditional autoregressive, such that the error in a census block group is conditioned on the error of its neighbors. Random error *v_i_* was defined with a normal distribution with 0 mean and variance *σ_v_*^2^. All statistical analyses were conducted using R version 4.3.1 (R Project for Statistical Computing). Models were fit using the R-INLA package^22^ and the BYM2 paramaterization.^23^ *P* values were 2-sided, and 95% bayesian credible intervals (BCI) were used for interpretation (α = .05).

Monitor buffers were defined based on the upper bound of the spatial extent of the monitor. To examine the effect of this choice, a sensitivity analysis was performed using the lower bound of the spatial extent in the models, only including census block groups if they were much closer to the monitor. Procedures for and results from the sensitivity analysis are described in eAppendices 3 and 4 in Supplement 1.

## Results

With a population of 329 725 481 US residents in 237 631 block groups (1 936 842 [.6%] American Indian and Alaska Native, 18 554 697 [5.6%] Asian, 40 196 302 [12.2%] Black, 60 806 969 [18.4%] Hispanic, 555 712 [.2%] Native Hawaiian and Other Pacific Islander, 196 010 370 [59.4%] White, 1 208 267 [.3%] some other race, and 10 456 322 [3.2%] 2 or more races), we analyzed the locations of 7771 EPA AQS monitors, 3672 (47.6%) of which were PM monitors, 1599 (20.6%) O_3_, 1074 (13.8%) SO_2_, 656 (8.4%) NO_2_, 364 (4.7%) Pb, and 346 (4.5%) CO. Census block group-level variables are summarized in the Table and stratified by whether that block group was monitored (yes or no) for each pollutant. For instance, the median (IQR) composition of Asian race in block groups that were not monitored for O_3_ was 0% (0-1.8%), while the composition in block groups that were monitored for O_3_ was 0.6% (0-5.2%). Most block groups were monitored for O_3_ (222 401 groups [93.6%]), PM (204 006 groups [85.8%]), NO_2_ (133 221 groups [56.1%]), and SO_2_ (129 799 groups [54.6%]), but most were not monitored for CO (100 797 groups [42.4%]) and Pb (14 945 groups [6.3%]). Most racial and ethnic compositions were significantly different, although this does not adjust for confounding by population size. We also modeled the number of monitors rather than a binary indicator of when there were any monitors.

**Table.** Monitored Populations by Pollutant

**Monitored for**	No. (%)	Participants, median (IQR), %								
		Population size	American Indian or Alaska Native	Asian^a^	Black or African American^a^	Hispanic or Latino	Native Hawaiian or Other Pacific Islander^a^	Two or more races^a^	White, median^a^	Some other race^a^
**Ozone**										
** No**	15 230 (6.4)	1141 (818-1591)	0 (0-0.1)	0 (0-1.8)	0.39 (0-7.4)	6.0 (1.0-23.2)	0 (0-0)	1.3 (0-4.1)	74.3 (41.8-90.0)	0 (0-0)
** Yes**	222 401 (93.6)	1263 (890-1754)	0 (0-0)	0.6 (0-5.2)	2.6 (0-14.2)	6.9 (1.3-22.1)	0 (0-0)	1.6 (0-4.3)	69.5 (36.7-88.5)	0 (0-0)
*** P* value**	NA	<.001	<.001	<.001	0.09	<.001	0.04	<.001	<.001	<.001
**Nitrogen dioxide**										
** No**	104 410 (43.9)	1189 (835-1661)	0 (0-0)	0 (0-1.9)	2.0 (0-14.3)	4.2 (0.4-13.6)	0 (0-0)	1.4 (0-4.1)	78.5 (52.6-91.9)	0 (0-0)
** Yes**	133 221 (56.1)	1306 (927-1802)	0-(0-0)	1.7 (0-8.2)	2.7 (0-13.3)	10.1 (2.3-30.4)	0 (0-0)	1.7 (0-4.5)	61.1 (26.9-84.3)	0.4 (1.8)
*** P* value**	NA	<.001	<.001	<.001	<.001	<.001	<.001	<.001	<.001	<.001
**Lead**										
** No**	222 686 (93.7)	1254 (883-1744)	0 (0-0)	0.4 (0-4.4)	2.3 (0-13.8)	6.4 (1.2-20.7)	0 (0-0)	1.55 (0-4.29)	71.5 (39.8-89.2)	0 (0-0)
** Yes**	14 945 (6.3)	1261 (902-1724)	0 (0-0)	4.2 (0-15.3)	2.9 (0-12.9)	18.4 (4.9-50.8)	0 (0-0)	1.8 (0-4.7)	38.7 (10.6-69.3)	0 (0-0)
*** P* value**	NA	0.22	<.001	<.001	<.001	<.001	<.001	<.001	<.001	<.001
**Carbon monoxide**										
** No**	136 834 (57.6)	1237 (863-1741)	0 (0-0)	0 (0-2.9)	2.4 (0-14.8)	6.0 (1.0-20.0)	0 (0-0)	1.4 (0-4.1)	72.7 (42.3-89.5)	0 (0-0)
** Yes**	100 797 (42.4)	1277 (914-1745)	0 (0-0)	1.7 (0-8.7)	2.4 (0-12.5)	8.2 (1.8-25.3)	0 (0-0)	1.78 (0-4.6)	65.4 (30.2-87.1)	0 (0-0)
*** P* value**	NA	<.001	<.001	<.001	0.093	<.001	0.036	<.001	<.001	<.001
**Sulfur dioxide**										
** No**	107 832 (45.4)	1252 (872-1763)	0 (0-0)	0 (0-2.9)	2.0 (0-13.3)	7.3 (1.4-24.0)	0 (0-0)	1.4 (0-4.1)	71.2 (39.3-88.9)	0 (0-0)
** Yes**	129 799 (54.6)	1258 (895-1728)	0 (0-0)	1.2 (0-7.2)	2.7 (0-14.1)	6.6 (1.2-20.7)	0 (0-0)	1.7 (0-4.5)	68.7 (35.1-88.4)	0 (0-0)
*** P* value**	NA	0.25	<.001	<.001	<.001	<.001	<.001	<.001	<.001	<.001
**Particulate matter**										
**No**	33 625 (14.2)	1248 (874-1749)	0 (0-0)	0 (0-2.69)	1.5 (0-11.6)	5.9 (1-18.8)	0 (0-0)	1.6 (0-4.3)	74.2 (46.0-89.9)	0 (0-0)
**Yes**	204 006 (85.8)	1256 (886-1742)	0 (0-0)	0.7 (0-5.4)	2.5 (0-14.1)	7.0 (1.3-22.8)	0 (0-0)	1.6 (0-4.3)	69.1 (35.3-88.4)	0 (0-0)
***P* value**	NA	0.14	<.001	<.001	<.001	<.001	<.001	0.78	<.001	0

^a^ All racial groups were collected as non-Hispanic or Latino. Comparisons were made between columns by pollutant (eg, ozone = no vs ozone = yes), with *P* values reported for 2-sided Kruskal-Wallis rank sum test. Analyses were performed with log transformations of racial and ethnic composition.

The geographic distribution of monitors and their spatial extents are shown by pollutant in Figure 1. O_3_ and PM had the fullest spatial extents, covering a large portion of the US in terms of area. NO_2_ covered population centers throughout the Northeast, Midwest, and California, but also throughout the Rocky Mountains. These monitors may be strategically placed near mines, oil drill sites, and refineries. The distributions of SO_2_ and CO monitors were otherwise similar to that of NO_2_. Pb had the sparsest distribution, covering no wide regions.

**Figure 2.**
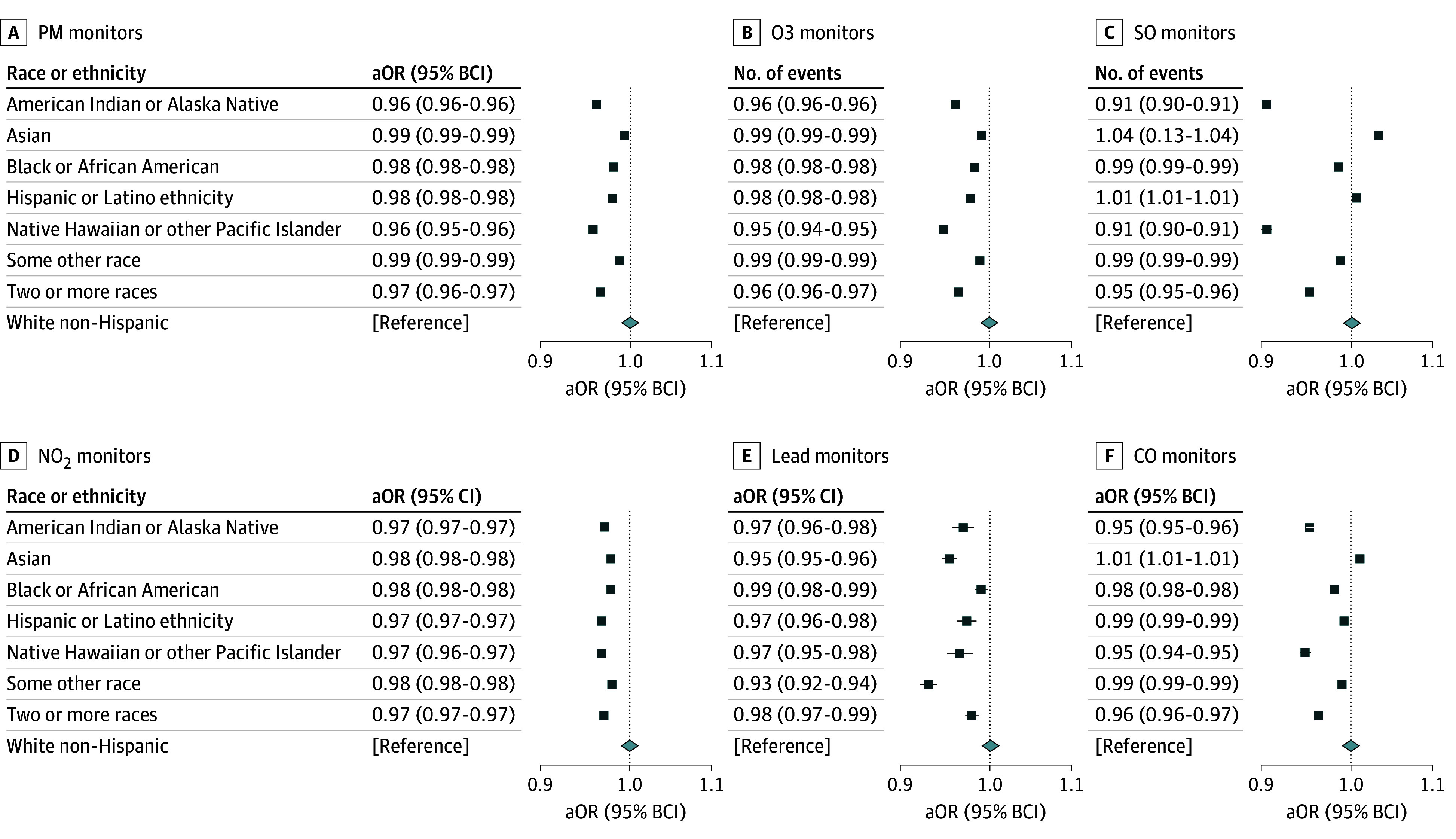
Model Results by Criteria Pollutant The adjusted odds ratios and 95% bayesian credible interval (BCI) are shown for each model. Each racial group was collected as non-Hispanic or Latino such that each racial and ethnic group was mutually exclusive. The White non-Hispanic or Latino group was used as the reference category.

Figure 2 shows the adjusted odds ratio and 95% BCI for each model. In the model of PM monitors, credible decreases in the number of PM monitors were associated with each racial group other than White and those of Hispanic of Latino ethnicity. The difference was largest, however, for those of Native Hawaiian and Other Pacific Islander race, American Indian or Alaska Native race, and those of 2 or more races. For a log-unit increase in each percentage, the number of PM monitors decreased by 4% with Native Hawaiian and Other Pacific Islander race (95% BCI, 0.95-0.96), 4% with American Indian or Alaska Native race (95% BCI, 0.96-0.96), and 4% with 2 or more races (95% BCI, 0.96-0.97).

The O_3_ model had similar results to the PM model, with credible decreases in the number of O_3_ monitors associated with the percentage in a census block group of all minoritized races and Hispanic or Latino ethnicity. For a log-unit increase in each percentage, the number of O_3_ monitors decreased by 5% with Native Hawaiian and Other Pacific Islander race (95% BCI, 0.96-0.97), 3% with Hispanic or Latino ethnicity (95% BCI, 0.97-0.97), 3% with American Indian or Alaska Native race (95% BCI, 0.97-0.97), and 3% with 2 or more races (95% BCI, 0.97-0.97).

The SO_2_ model had slightly different results from the other models, with greater magnitude. For a log-unit increase in each percentage, the number of SO_2_ monitors decreased by 9% with Native Hawaiian and Other Pacific Islander race (95% BCI, 0.90-0.91), 9% with American Indian or Alaska Native race (95% BCI, 0.90-0.91), and 5% with 2 or more races (95% BCI, 0.95-0.96). Unlike the previous 2 models, notable increases in number of monitors were also observed. The number of SO_2_ monitors increased by 4% with Asian race (95% BCI, 1.03-1.04), and only 1% with Hispanic or Latino ethnicity (95% BCI, 1.01-1.01).

Similar to PM and O_3_, in the NO_2_ model, credible decreases in number of monitors were seen across all racial groups other than White and Hispanic or Latino ethnicity. These differences were relatively modest, associated with decreases of 2% to 3%. Both Native Hawaiian and Other Pacific Islander race and Hispanic or Latino ethnicity were associated with the largest decreases of 3% (Native Hawaiian and Other Pacific Islander: 95% BCI, 0.91-0.94, Hispanic or Latino: 95% BCI, 0.91-0.94).

The Pb model had fairly similar results to PM. However, the effects of Asian race and some other race were associated with a larger decrease in Pb monitors than any other model. For a log-unit increase in each percentage, the number of Pb monitors decreased by 7% with some other race (95% BCI, 0.93-0.93), 5% with Asian race (95% BCI, 0.95-0.96), 6% with Hispanic or Latino ethnicity (95% BCI, 0.92-0.96), and 3.5% with Native Hawaiian and Other Pacific Islander race (95% BCI, 0.95-0.98).

CO results were relatively consistent with the other models. For a log-unit increase in each percentage, the number of CO monitors decreased by 5% with Native Hawaiian and Other Pacific Islander race (95% BCI, 0.94-0.95), 5% with American Indian or Alaska Native race (95% BCI, 0.95-0.96), 4% with 2 or more races (95% BCI, 0.96-0.97), and 2% with Black or African American race (95% BCI, 0.98-0.98). Additionally, Asian race was associated with a 1% increase in the number of CO monitors (95% BCI, 1.01-1.01).

## Discussion

In this cross-sectional study of EPA regulatory monitors in the US, we found that the spatial distribution of monitors was not equitable across racial and ethnic groups. AQS data, therefore, may not be equitably representative of air quality by race and ethnicity. In particular, our results suggested systemic racial and ethnic monitoring disparities for PM, O_3_, NO_2_, and Pb. Monitoring disparities also existed for SO_2_ and CO, particularly for Native Hawaiian and Other Pacific Islander and American Indian or Alaska Native populations. Although the magnitude of most effects was somewhat small, the change in number of monitors was also relative to a small increase in racial and ethnic composition—from 1% to 2.7%, on a nonlinear scale.

In most models, block groups with a greater proportion of Native Hawaiian and Other Pacific Islander race or American Indian or Alaska Native race were most likely to be affected. Population concentrations were highest for Native Hawaiian and Other Pacific Islander individuals in Hawaii, Utah, Washington, and California.^24^ Within these states, monitor coverage may be relatively poor for this population. Rurality may also reduce monitor coverage for American Indian or Alaska Native populations, but the disparity existed after accounting for population size.

At a national scale, even small inequities in air quality monitoring could have profound impacts on data bias. We focused on monitoring disparities from 2019 to 2024, but historical variation in monitor locations may further bias AQS data (eFigure 5 in Supplement 1). Compared with monitored areas, undermonitored or unmonitored areas may have better or worse air pollution, but the air quality cannot be ascertained without direct measurement. This measurement error could result in bias, particularly if uncertainty in estimates vary across the health outcome of interest in a study.

Our results somewhat contrast with the work of Miranda et al,^10^ which focused on both undermonitoring and poor air quality in the contiguous US from 2005 to 2007. This may be due to different definitions of monitoring, as undermonitoring was defined as having a number of monitors disproportional to the population within a county, given nonattainment status. We did not observe greater PM and O_3_ monitoring for Hispanic or Latino ethnicity or Black or African American race. Rather, both groups were associated with slightly fewer PM and O_3_ monitors per capita at the block group level. While the average county in our study area was approximately 2973 km^2^ in size, regulatory monitors provide samples for areas as small as 0.03 km^2^, with neighborhood (50.27 km^2^) being the most common sample area. The average area of a census block group is 39 km^2^. Our definition considered these smaller administrative units, as monitor coverage may vary significantly within counties and across demographic characteristics, especially where racial residential segregation is present. Compared with the PM and O_3_ results, we observed that each racial and ethnic group had larger disparities for at least 2 other criteria pollutants. Pb, SO_2_, and CO are also more difficult to measure using satellite imagery than PM, O_3_, and NO_2_.

Regulatory monitors remain an essential source of air pollution data, despite technological and methodological advances in environmental monitoring. Low-cost sensors provide precise but sometimes inaccurate estimates for particulate matter,^25^ and the spatial extent of these monitors is sparser than the AQS in most areas of the US.^25,26^ By contrast, satellite data have wide spatial coverage and can measure particulate matter as well as trace gases.^27^ However, remote sensing is limited at night and when clouds or smoke are present in the atmosphere.^27,28^ Additionally, orbiting satellites observe a location about once a day, although geostationary satellites may continuously measure an area.^27^ Furthermore, satellites sample a vertical cross-section of the atmosphere and do not capture surface-level estimates.^27^ Ground data may still be needed for validation.^27^ Depending on the objective, researchers and public health officials may find that integrating multiple sources of air pollution data suits their needs. Uncertainty may be reduced, even if it cannot be eliminated, and measures of uncertainty should be reported where possible.

### Limitations

This study had limitations. This work is cross-sectional in nature, and so causal statements about the effect of demographic composition on number of monitors cannot be made. However, as our interest is in disparities that exist regardless of cause, our conclusions about associations between demographic composition and number of monitors were not necessarily weakened by this.

We used ACS 5-year estimates for racial and ethnic composition, which classifies individuals into the minimum set of categories defined by the US Office of Management and Budget. Substantial racial and ethnic variability exists within these categories, but more granular groupings were not available, so census categories were used. This helped preserve statistical power, but also allows for greater comparability with existing research.

Although most AQS monitors record continuously (eTable 2 in Supplement 1), all Pb and 42% of PM monitors record intermittently. More detailed analyses of disparities in data quality may consider recording strategy, but also missingness and quality control.

## Conclusions

The findings of this study suggest that EPA regulatory monitor data may not adequately capture air quality for some marginalized race and ethnicity groups, particularly Native Hawaiian and Other Pacific Islander and American Indian or Alaska Native populations. The consequences of inadequate monitoring on air quality estimates for these communities should be investigated further. The location of new monitors could be optimized to improve both spatial sampling and inequities. Integration of multiple data sources may aid in filling monitoring gaps across race and ethnicity.

## Notes

Supplement 1. **eAppendix 1. **Coefficient interpretation **eFigure 1. **Racial and ethnic composition visualized at the county level using Fisher class intervals **eFigure 2. **Block group–level population size **eTable 1. **Measurement scales and missingness by pollutant **eFigure 3. **Outcome variable creation **eAppendix 2. **Alternative strategies for measuring the outcome **eFigure 4. **Identifying confounders with a directed acyclic graph **eAppendix 3. **Sensitivity analysis procedures **eAppendix 4. **Sensitivity analysis results **eFigure 5. **Monitor locations over time **eTable 2. **Recording strategies by pollutant
eReferences.

Supplement 2.
Data Sharing Statement
