# Supplementary material for: Racial and Ethnic Disparities in Regulatory Air Quality Monitor Locations in the US
Source: JAMA Netw Open. 2024 Dec 4;7(12):e2449005. doi: 10.1001/jamanetworkopen.2024.49005 (PMC11618472; doi:10.1001/jamanetworkopen.2024.49005)
Supplement: Supplement 1. — eAppendix 1. Coefficient interpretation eFigure 1. Racial and ethnic composition visualized at the county level using Fisher class intervals eFigure 2. Block group–level population size eTable 1. Measurement scales and missingness by pollutant eFigure 3. Outcome variable creation eAppendix 2. Alternative strategies for measuring the outcome eFigure 4. Identifying confounders with a directed acyclic graph eAppendix 3. Sensitivity analysis procedures eAppendix 4. Sensitivity analysis results eFigure 5. Monitor locations over time eTable 2. Recording strategies by pollutant eReferences. [file jamanetwopen-e2449005-s001.pdf]

Supplemental Online Content

Kelly BC, Cova TJ, Debbink MP, Onega T, Brewer SC. Racial and ethnic disparities in regulatory air quality monitor locations in the US. *JAMA Netw Open*. 2024;7(12):e2449005. doi:10.1001/jamanetworkopen.2024.49005

eAppendix 1. Coefficient interpretation
eFigure 1. Racial and ethnic composition visualized at the county level using Fisher class intervals
eFigure 2. Block group–level population size
eTable 1. Measurement scales and missingness by pollutant
eFigure 3. Outcome variable creation
eAppendix 2. Alternative strategies for measuring the outcome
eFigure 4. Identifying confounders with a directed acyclic graph
eAppendix 3. Sensitivity analysis procedures
eAppendix 4. Sensitivity analysis results
eFigure 5. Monitor locations over time
eTable 2. Recording strategies by pollutant
eReferences.

This supplemental material has been provided by the authors to give readers additional information about their work.

© 2024 Kelly BC et al. *JAMA Network Open*.

eAppendix 1. Coefficient interpretation

Race and ethnicity were collected as percentages of each Census-classified race and ethnicity in a Census block group. Each race and ethnicity variable was log-transformed due to string right-skewed distributions. When modeled in our regression, this means that the variables were centered on 0 after the log transformation, making the reference value $e^0 = 1$ for all groups. The model coefficients are given relative to a log-unit increase from the reference value, or $e^1 - e^0 = 2.72 - 1 = 1.72$. In other words, the full interpretation of coefficients might be:

$$\beta_{Asian} = -0.05$$

$$\mathrm{e}^{-0.05} = 0.95$$

Relative to an area with a population of 1% Asian residents, a 1.72% increase in the percent of Asian residents is associated with 5% fewer monitors. The reference population would be composed of 1% Asian residents, but also 1% each of AIAN, Black or African American, NHPI, Two or More races, some other race, and Hispanic or Latino ethnicity. In other words, the reference population is composed of 93% non-Hispanic White residents and 1% each of every other group.

eFigure 1. Racial and ethnic composition visualized at the county level using Fisher class intervals

County-level racial and ethnic composition with the groups used in our study are mapped in eFigure 1. Fisher class intervals are used, as these maximize differences between class intervals according to the distribution.

Block group–level population is shown in eFigure 2. Decile intervals are used to show the distribution, which is right-skewed.

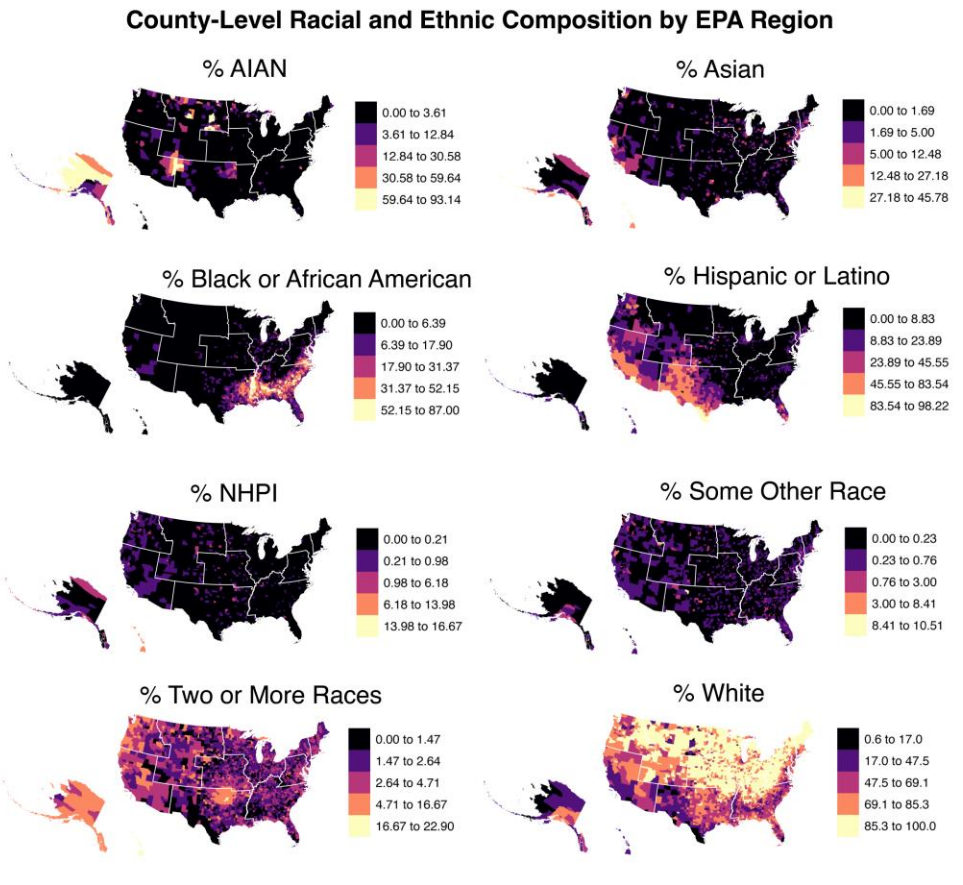

eFigure 1. Racial and ethnic composition are visualized at the county level using Fisher class intervals.

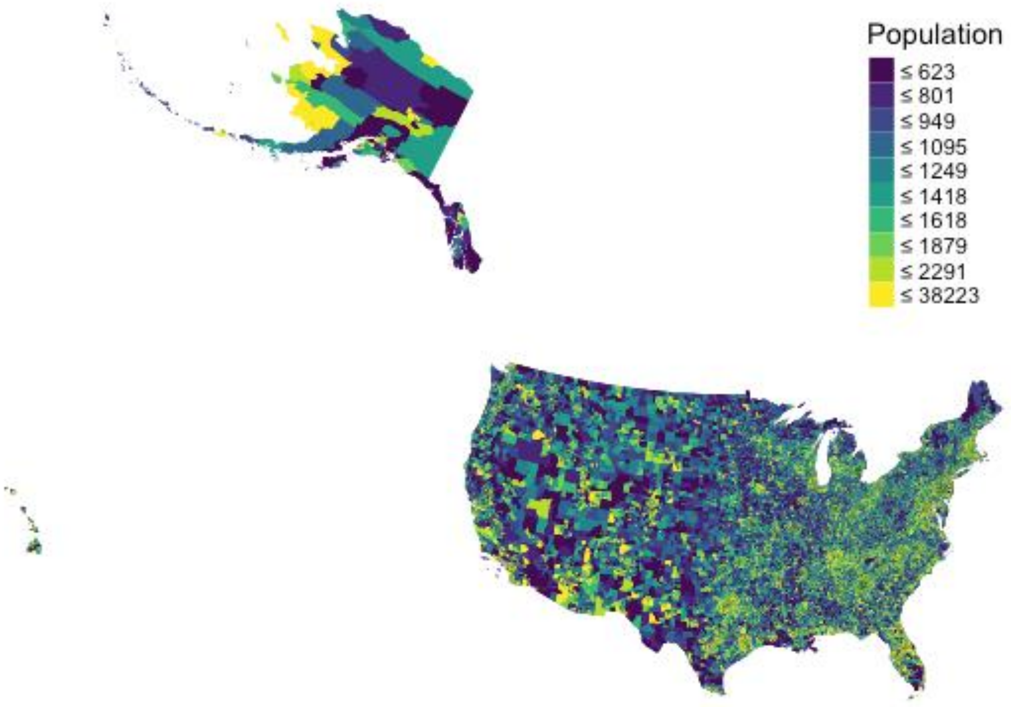

eFigure 2. Block group–level population size

eFigure 2. Block group–level population size

Not all monitors had a measurement scale listed in the AQS dataset (eTable 1). Rather than exclude these monitors, we imputed the measurement scale based on the modal scale for that pollutant. Alternative strategies may have included exclusion of missing data, single imputation with multinomial regression, and multiple imputation with multinomial regression. Exclusion would only be appropriate when missingness occurs completely at random, which is unlikely and difficult to prove. With any imputation method, we impose our own assumptions about which variables would best account for structure in missingness, if missing at random (not *completely* at random). With modal imputation, a single imputation strategy, we use information from related observations based on criteria pollutant.

eTable 1. Measurement scales and missingness by pollutant		**Measurement Scale**					
		Microscale	Middle	Neighborhood	Urban	Regional	Missing
**CO**	N (%)	74 (21.4)	33 (9.5)	151 (43.6)	37 (7.8)	23 (6.6)	38 (11.0)
**NO_2**	N (%)	66 (10.1)	37 (5.6)	261 (39.8)	122 (18.6)	51 (7.8)	119 (18.1)
**O_3**	N (%)	4 (0.3)	19 (1.2)	581 (36.3)	502 (32.4)	298 (18.6)	195 (12.2)
**Pb**	N (%)	38 (10.4)	86 (23.6)	132 (36.3)	6 (1.6)	0 (0.0)	102 (28.0)
**PM**	N (%)	103 (10.7)	173 (4.7)	2224 (60.6)	392 (10.7)	233 (6.3)	547 (14.9)
**SO_2**	N (%)	16 (1.5)	71 (6.6)	534 (49.7)	143 (13.3)	70 (6.5)	240 (22.3)

© 2024 Kelly BC et al. *JAMA Network Open*.

eFigure 3. Outcome variable creation

We used several procedures to create our outcome variable (eFigure 3), which was the number of monitors sampling a Census block group's population for each criteria pollutant. First, Air Quality System monitor locations were collected as a csv from the EPA's AirNow webpage. The criteria pollutant sampled by each monitor was examined, and only parameter codes for criteria pollutants were included. Using the latitude and longitude fields, the monitor dataframe was converted to a spatial point feature. The World Geodetic System 1984 (WGS84) coordinate reference system was used, and the point feature was transformed to the Albers Equal Area projection.

Next, the 2020 Block Group Centers of Population dataset was obtained from the U.S. Census Bureau as a csv file. The dataframe was converted to a spatial point feature, also using the WGS84 coordinate reference system and the Albers Equal Area projection.

To link the monitor locations to the population centroids, we created a buffer around each monitor. The size of the buffer was determined based on the measurement scale of the monitor (micro-scale: 100 m, middle scale: 100 m–0.5 km, neighborhood scale: 0.5–4 km, urban scale: 4 km–50 km, regional scale: "tens to hundreds of kilometers," 50–150 km used[1]). The upper extent of the monitors was used for our model, but a sensitivity analysis (see Supplement 6) was conducted with the lower extent of the monitors. The monitors were split into five shapefiles, one for each measurement scale. Based on the scale values (100 m, 0.5 km, 4 km, 50 km, 150 km), buffers were created around all monitors.

The buffer polygons were then split into six spatial features, one for each pollutant. For each measurement scale, a spatial join was performed to identify which buffers for each

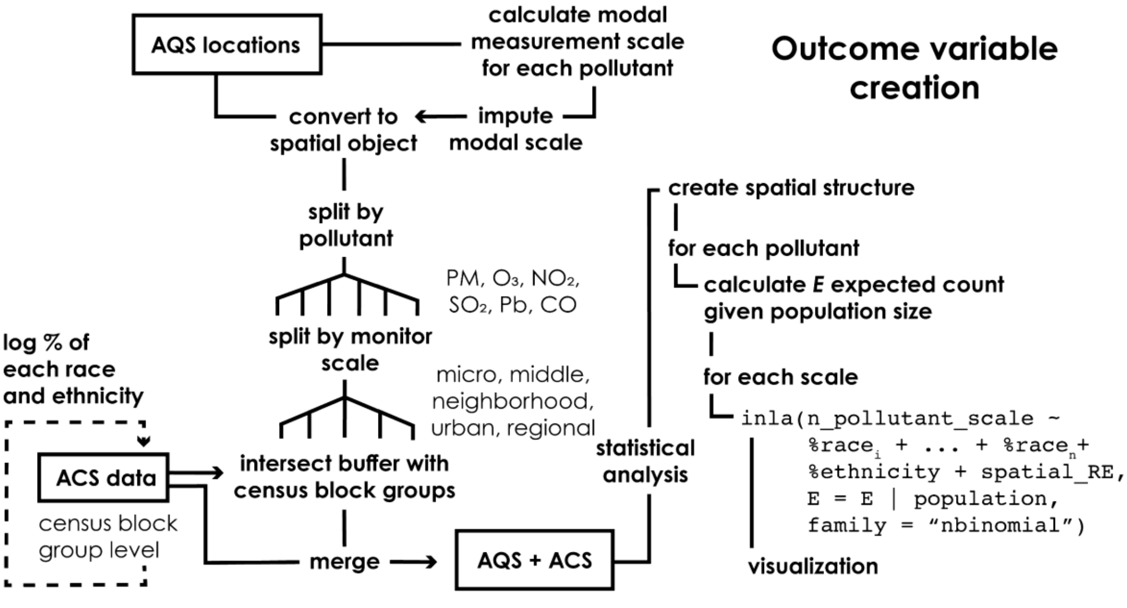

eFigure 3. Outcome variable creation schematic

pollutant contained the population centroids. For each pollutant, the resultant

© 2024 Kelly BC et al. *JAMA Network Open*.

dataframes were combined (rbind). The number of times a Census block group's FIPS code appeared in the combined dataframe indicated the number of monitors for that pollutant measuring that area's population. These values were merged with the ACS demographic data.

eAppendix 3. Alternative strategies for measuring the outcome
We considered several alternative strategies for defining the outcome (who is monitored), including distance-based methods and kriging, but were determined to be too reductive and less interpretable. Additionally, population monitoring based on the scaled determined by the EPA more direct policy and population health implications.

Monitors sample at different scales and therefore measure different things. Population A may live 0.5 hm from a microscale monitor, but these are intended to measure pollution which may be localized to a particular area. They may also live 3 km from a neighborhood scale monitor, which is intended to capture trends in concentration typical to their location. Neither kriging nor proximity-based approaches would not allow us to consider this nuance.

Distance to nearest monitor was considered, as being closer to a monitor may mean your exposure is more closely related to what was recorded. However, we also argue that more monitoring reduces measurement error, and this would be lost without considering the effect of multiple monitors. If Population A lived 1 km from one monitor and Population B lived 1 km from one monitor 1.1 km from three monitors, the outcome would be identical for both populations.

In an earlier iteration of the study, we created a kriged surface of monitor density. Unlike the proximity approach, this does consider the effect of multiple monitors. However, this would still ignore the measurement scale. We also determine that the methods and results were less interpretable than a generalized linear model.

Many factors may affect how reliably a monitor samples a given area. Without finer scale monitor data, we can't know if the monitor provides a good approximation of exposure in the measurement area (and there is probably temporal variation in how well it approximates). There is almost certainly spatial heterogeneity within these areas. We assume the choice in monitor placement and measurement scale are informed by spatial and temporal heterogeneity within the area. While this is a fairly large assumption, it is grounded in EPA regulatory monitoring guidelines — we can identify gaps where, by the EPA and local authority's own measures, we know little to nothing about air quality.

© 2024 Kelly BC et al. *JAMA Network Open*.

eFigure 4. Identifying confounders with a directed acyclic graph
Well-established associations among the variables were delineated (eFigure 4).[1–4] Variables were selected based on EPA monitoring objectives:

1. Determine the highest concentration expected to occur in the area covered by the network. (**industrial activity, human activity, pollution sources**)
2. Measure typical concentrations in areas of high population density. (**population size, urban environment**)
3. Determine the impact of significant sources or source categories on air quality. (**industrial activity, human activity, pollution sources**)
4. Determine background concentration levels. (**industrial activity, human activity, pollution sources**)
5. Determine the extent of regional pollutant transport among populated areas; and in support of secondary standards. (**urban environment, human activity**)
6. Measure air pollution impacts on visibility, vegetation damage, or welfare-based impacts. (**population size, urban environment, pollution sources**)

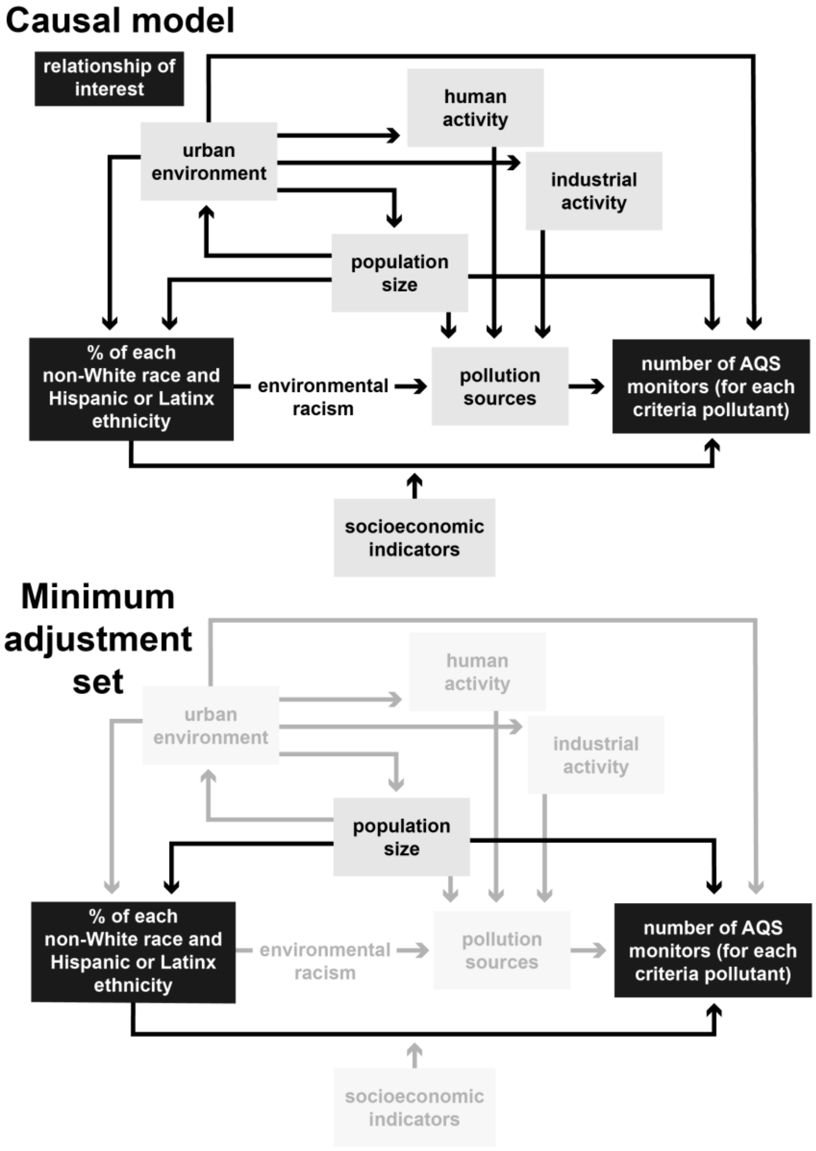

eFigure 4. Directed Acyclic Graph

The minimal adjustment set is derived using the **adjustmentSets** function in **dagitty**, an R package for graphical analysis of structural causal models. This function enumerates sets of covariates that allow for unbiased estimation of causal effects from observational data.

© 2024 Kelly BC et al. *JAMA Network Open*.

eAppendix 3. Sensitivity analysis procedures

When constructing buffers based on the upper extent of each monitor's measurement scale (100 m, 0.5 km, 4 km, 50 km, 150 km), we also constructed buffers based on the lower extent (1 m, 100m, 0.5 km, 4 km, 50 km). The same procedures were used as above (Supplement 2. Measurement scale imputation).

eAppendix 4. Sensitivity analysis results

When restricting the monitor measurement scales to the lower bound, most effects in each model followed a similar direction with some exceptions. The effect of Asian race reversed direction in the CO and SO2 models. The effect of Hispanic or Latino ethnicity also reversed in the SO2 model, and the effect of Some Other Race was amplified. Across all effects, confidence intervals widened in every model, but these overlapped with the initial confidence intervals in most cases. In the Pb model, confidence intervals widened substantially, such that most effects were not credible, with the exception of the effect of Asian race. Using the lower bound of measurement scales, only 394 Census block groups (0.2%) were considered monitored for Pb. The magnitude of most effects changed only slightly in the models of CO, PM, and O3, but the magnitude was amplified in most effects of the NO2 model.

© 2024 Kelly BC et al. *JAMA Network Open*.

eFigure 5. Monitor locations over time

eFigure 5 shows changes in monitor density throughout the history of EPA air quality monitoring, beginning in 1957. All monitors are represented with a single circle, regardless of measurement scale. The Clean Air Act was first passed in 1963 and later amended in 1977 and 1990. Pb monitors were the first to be placed, and many CO, SO_2, O_3, and NO_2 monitors were added in the early-to-mid 1970s. PM and O_3 are monitored more heavily beginning in the late 1980s.

See figure 5 here:
https://drive.google.com/file/d/1ynIImn0Tq55mVVWMD04dKyTh9dnFLm2G/view?usp=share_link

eFigure 5. Monitor locations over time

© 2024 Kelly BC et al. *JAMA Network Open*.

eTable 2. Recording strategies by pollutant

Of the 7,771 monitors studied in this analysis, 4,811 (61.9%) monitored continuously, 1,781 (22.9%) monitored intermittently, and 1,113 (14.3%) did not have a recording strategy listed (eTable 2). Of the monitors without a missing strategy, all O_3 monitors were continuous, all Pb monitors were intermittent, and all CO were continuous. Nearly all SO_2 and NO_2 monitors were continuous. PM is the only pollutant with a substantial split between continuous and intermittent monitors.

eTable 2. Recording strategies by pollutant		**Recording Strategy**		
		Continuous	Intermittent	Missing
		N = 4811	N = 1787	N = 1113
**PM**	N (%)	1785 (48.6)	1539 (41.9)	348 (9.5)
**O_3**	N (%)	1369 (85.6)	0 (0)	230 (14.4)
**SO_2**	N (%)	806 (75)	4 (0.4)	264 (24.6)
**NO_2**	N (%)	547 (83.4)	2 (0.3)	107 (16.3)
**Pb**	N (%)	0 (0)	242 (66.5)	122 (33.5)
**CO**	N (%)	304 (87.9)	0 (0)	42 (12.1)

eReferences

1. United States Environmental Protection Agency. 6.0 Monitoring Network Design. In: *Quality Assurance Handbook for Air Pollution Measurement Systems*. Vol II. ; 2013:4-5.
2. He H, Schäfer B, Beck C. Spatial heterogeneity of air pollution statistics in Europe. *Sci Rep*. 2022;12(1):12215. doi:10.1038/s41598-022-16109-2
3. Xie X, Semanjski I, Gautama S, et al. A Review of Urban Air Pollution Monitoring and Exposure Assessment Methods. *ISPRS Int J Geoinf*. 2017;6(12):389. doi:10.3390/ijgi6120389
4. Lane HM, Morello-Frosch R, Marshall JD, Apte JS. Historical Redlining Is Associated with Present-Day Air Pollution Disparities in U.S. Cities. *Environ Sci Technol Lett*. 2022;9(4):345-350. doi:10.1021/acs.estlett.1c01012

© 2024 Kelly BC et al. *JAMA Network Open*.
