# Supplementary material for: Racial and Ethnic Disparities in Regulatory Air Quality Monitor Locations in the US
Source: JAMA Netw Open. 2024 Dec 4;7(12):e2449005. doi: 10.1001/jamanetworkopen.2024.49005 (PMC11618472; doi:10.1001/jamanetworkopen.2024.49005)
Supplement: Supplement 2. — Data Sharing Statement [file jamanetwopen-e2449005-s002.pdf]

# Data Sharing Statement

Kelly. ZXC Racial and Ethnic Disparities in Regulatory Air Quality Monitor Locations in the US. *JAMA Netw Open*. Published December 04, 2024. doi:10.1001/jamanetworkopen.2024.49005

**Data**
**Data available:** Yes
**Data types:** Data (not involving human participants)
**How to access data:** Data will be hosted in a GitHub repository: github.com/brenna-c-kelly/aqs_racial_inequity
**When available:** With publication

**Supporting Documents**
**Document types:** Statistical/analytic code
**How to access documents:** The R code for this analysis is freely available in a GitHub repository: github.com/brenna-c-kelly/aqs_racial_inequity
**When available:** With publication

**Additional Information**
**Who can access the data:** Anyone requesting the data
**Types of analyses:** For any purpose
**Mechanisms of data availability:** Without investigator support
